# The Effect of Integration of Family Planning Into HIV Services on Contraceptive Use Among Women Accessing HIV Services in Low and Middle-Income Countries: A Systematic Review

**DOI:** 10.3389/fgwh.2022.837358

**Published:** 2022-02-24

**Authors:** Tallulah Grant-Maidment, Katharina Kranzer, Rashida A. Ferrand

**Affiliations:** ^1^London School of Hygiene and Tropical Medicine, London, United Kingdom; ^2^Biomedical Research and Training Institute, Harare, Zimbabwe

**Keywords:** family planning, HIV, integration, contraception, safer conception, systematic review

## Abstract

There is substantial unmet need for family planning (FP) among women living with HIV (WLHIV), leading to unintended pregnancies and may contribute indirectly to increasing the risk of transmission of HIV. This review aims to determine whether integration of FP into HIV testing and care results in increased use of contraception, a reduction in unmet need for FP, improved use of safer conception methods and a reduction in unintended pregnancies in low and middle-income countries. A systematic review was undertaken incorporating studies from PubMed, EMBASE, CINAHL, Web of Science and Global Health, the International AIDS Society Abstract Archive, the World STI & HIV Congress Abstract Archive and the Conference on Retroviruses and Opportunistic Infections Abstract Archive published between 2016 and 2021, updating previous systematic reviews. After screening, 13 studies were included, 11 conducted in sub-Saharan Africa and 2 in India. The primary outcome of the review was contraceptive uptake and secondary outcomes included unmet need for FP, safer conception and unintended pregnancy. Integrated FP-HIV facilities were found to increase dual contraceptive use by at least 8% in five studies and modern contraceptive use by at least 8% in four studies. Findings from two studies suggested integration decreased the unmet need for contraception. Limited data prevented a conclusion from being drawn regarding whether integration increases safer conception. There was no evidence of integration reducing unintended pregnancies. The median quality score of studies was 3/9. Overall, integrated facilities have the potential of improving reproductive health of women accessing HIV services in LMICs. FP may be enhanced by including a safer conception component for WLHIV.

**Systematic Review Registration:**

https://www.crd.york.ac.uk/prospero/display_record.php?ID=CRD42021251008, identifier: CRD42021251008.

## Introduction

The HIV epidemic continues to present an immense global public health challenge with ~37.7 million individuals living with HIV in 2020, two thirds being in sub-Saharan Africa (1). The global scale-up of antiretroviral therapy (ART) has substantially reduced both morbidity and mortality (2). To deliver ART to large numbers of people, many HIV care programmes in low and middle income countries (LMIC) with generalized HIV epidemics use a public health approach using standardized simplified treatment protocols and decentralized service delivery (3). While this approach has enabled the scale-up of ART in overwhelmed health systems, broader health needs of people with HIV are often not addressed.

An example is family planning (FP), which is often provided through separate vertical programmes (4). FP enables women to make decisions regarding the number, timing and spacing of pregnancies and enables better reproductive health outcomes through education about birth spacing and provision of contraception (5). Successive births separated by <2 years are associated with 45% higher infant mortality than with births are separated by two or more years (6, 7). Unintended pregnancies carry a greater risk of poorer health outcomes than planned pregnancies such as low infant birth weight and poor maternal mental health (8, 9). In addition, unintended pregnancies in women living with HIV (WLHIV) are associated with late presentation for antenatal care and therefore delayed access to ART for prevention of mother-to-child transmission (PMTCT) and reduced adherence to ART, both of which increase the risk of mother-to-child HIV transmission (10).

WLHIV have a much higher unmet need for FP than HIV negative women (11). In sub-Saharan Africa, 66–92% of WLHIV reported not wanting another child and yet only 20–43% of these women were utilizing a form of contraception (12). The unmet need for FP among WLHIV is due to multiple factors including social marginalization and poverty (4). Young women and adolescents living with HIV face further barriers including restrictive policies and stigma, preventing access to sexual and reproductive health services (13).

Reducing the unmet need for contraception in WLHIV has the potential to not only improve reproductive health outcomes but also to reduce the risk of mother-to-child HIV transmission (14). Safer conception interventions embedded in family planning services, such as education and counseling, ensuring viral suppression in the mother and/or her partner, timed condom-less intercourse, intravaginal or intrauterine insemination and pre-exposure prophylaxis (PrEP) initiation for HIV-negative partners, may reduce the risk of vertical HIV transmission as well as horizontal transmission when partners are sero-discordant (15).

Integration of FP services into HIV services has been proposed as a method to decrease the unmet need for FP among WLHIV in LMICs (16). Integration of services may improve convenience for patients and strengthen the health systems (14). Three previous systematic reviews have been conducted between 2009 and 2017 on the integration of FP into HIV care (17–19). Spaulding et al. considered integration to be feasible and effective (18). They found some evidence of integration increasing contraceptive use, however, not enough studies reported on this outcome to conclude an effect (18). Wilcher et al. later concluded that while an association between integrated services and increased contraceptive use exists, average study quality was weak (a mean of 3.4 out of 9) (19). The latest review was conducted by Haberlen et al. and included studies up to February 2016 (17). The investigators reported integration increased the use of modern contraception methods and three studies reported an association between integration and dual method use (17). However, limited evidence prevented an evaluation for unintended pregnancy or unmet need for FP (17). Previous reviews did not include safer conception, defined as interventions to reduce the risk of mother-to-child HIV transmission as an outcome of interest. In September 2015, the World Health Organization (WHO) released guidelines recommending all pregnant WLHIV should be provided with lifelong ART, regardless of clinical or immunological status (20). This approach further minimized the risk of mother-to-child transmission of HIV during pregnancy (20). This coincided with an increase in the accessibility of pre-exposure prophylaxis (PrEP) globally (21). Therefore, since the previous review, safer conception has become an even more relevant outcome in relation to provision of FP.

We conducted a systematic review to update evidence presented in previous reviews and investigate whether integration of FP and HIV services increases uptake of contraception. Other examined outcomes included unmet need for FP, safer conception practices and unintended pregnancy.

## Methods

### Search Strategy

A systematic review was conducted using PRISMA guidelines and the review was registered with PROSPERO. PubMed, EMBASE, CINAHL, Web of Science and Global Health databases were searched in June 2021. The database search employed a summation of three concepts: family planning, HIV and integration, guided by previous reviews. Terms searched for family planning included “family planning,” “contraception,” “birth control,” “safer conception,” “planned parenthood,” “birth spacing,” and “birth prevention.” HIV terms included “HIV,” “Human immunodeficiency virus,” “AIDS,” and “acquired immunodeficiency syndrome.” Concept three included the terms “integrated,” “integration,” and “linkage.” The search was limited to articles in English published between March 2016 and May 2021 as the previous review included studies up to February 2016. Three conference abstract archives: International AIDS Society, the World STI & HIV Congress and the Conference on Retroviruses and Opportunistic Infections (CROI) were also searched utilizing a keyword search. The Cochrane Central Register of Controlled Trials was searched to identify any unpublished or ongoing trials and references from the bibliographies of the included studies were similarly examined.

### Eligibility Criteria

Studies with women of reproductive age (15–49 years) accessing HIV testing or care services in any LMIC defined according to the World Bank were included (22). HIV testing and care interventions included provider-initiated and client-initiated testing and counseling, treatment services and PMTCT services. The definition of FP interventions for inclusion in this review included contraception and safer conception education and counseling, provision of contraception methods and referrals for contraception methods not available at the point of the HIV service (including termination of pregnancy). Studies with no detail of the FP intervention were excluded. Studies without a comparator were not eligible for inclusion. Suitable comparators included control groups, comparison groups and before/after designs. Integration was defined as any relevant FP intervention (as defined above) specifically incorporated into any HIV testing and care service (as defined above). This could be, delivered by the same provider in one appointment, on the same site or through an enhanced referral service. The primary outcome of the review was contraception use. This was defined as use or desire to use any contraceptive method. Categories within this outcome included dual method contraception and modern contraception. Use of condoms in addition to another contraceptive is defined as dual contraception use (23). The definition of modern contraceptive methods is dependent on national guidelines (24). All definitions of modern contraception include contraceptive injectables, implants, IUDs, oral contraceptives and vaginal rings. However, while some definitions include condoms, others exclude them due to the inconsistency of use (24). The secondary outcomes in this review were unintended pregnancy, unmet need for FP and safer conception. Unintended pregnancy was defined as when an infant was either not desired or conceived earlier than desired (25). The unmet need for FP was defined as not desiring a pregnancy in a designated period of time and yet not using the contraceptives required to prevent a pregnancy or WLHIV wanting to conceive but not receiving the support to do so safely (26). Finally, safer conception was defined as any intervention to minimize the risk of HIV transmission from mother to child in WLHIV desiring a pregnancy for example through discussions with a healthcare professional or the practicing of safer conception methods (15). Experimental or observational designs were included and qualitative studies, case report, case series and studies proposing models were excluded.

### Study Selection and Data Extraction

The results of the search strategy were exported into EndNote X9 and one reviewer screened search results against the exclusion criteria first by their titles, then abstracts and full-texts. Duplicates were deleted after examination of their titles, authors and date of publication. Following full-text screening, data was extracted into Excel from all eligible studies. Extracted data included the article title, authors, date, study design, location, study duration, target population, type of FP intervention, type of HIV care, outcomes and results. Data extraction was performed twice to minimize transcription error.

### Assessment of Study Quality

The same 9-item tool, used to assess study quality in the three previous systematic reviews, was used for continuity (27). Spaulding et al. adapted this scale from HIV behavioral intervention reviews (18). One point was allocated for each of the criteria met. The criteria included: pre/post-intervention data availability, control or comparison group, cohort analysis, comparison groups being equivalent at baseline on sociodemographic characteristics, comparison groups being equivalent at baseline on outcome measures, random assignment, participants being randomly selected for assessment, control for potential confounders and having a follow-up rate ≥75%. A score of ≥7/9 was deemed a high-quality study, scores between 4 and 6/9 were considered to carry risk of bias, and those scoring <4/9 were considered weak quality studies and thus at high risk of bias.

Cohort and case-control studies underwent an additional assessment using the Newcastle-Ottawa Scale, which utilizes the allocation of stars in three categories: selection, comparability and outcome. Studies attaining ≥7 stars out of a possible 9 were considered high-quality, those scoring between 4 and 6 stars were considered to be at high risk of bias and those with 0–3 stars had a very high risk of bias.

### Data Analysis

All included studies were categorized by whether the outcome assessed contraceptive uptake, unintended pregnancy, unmet need for FP or safer conception. Data on these outcomes was extracted into Excel and analyzed. A meta-analysis was considered but given the studies were very heterogeneous with respect to FP intervention, HIV service and the extent of integration, this was not feasible. Instead, a descriptive analysis and narrative synthesis were conducted.

## Results

### Study Characteristics

**Figure 1:**
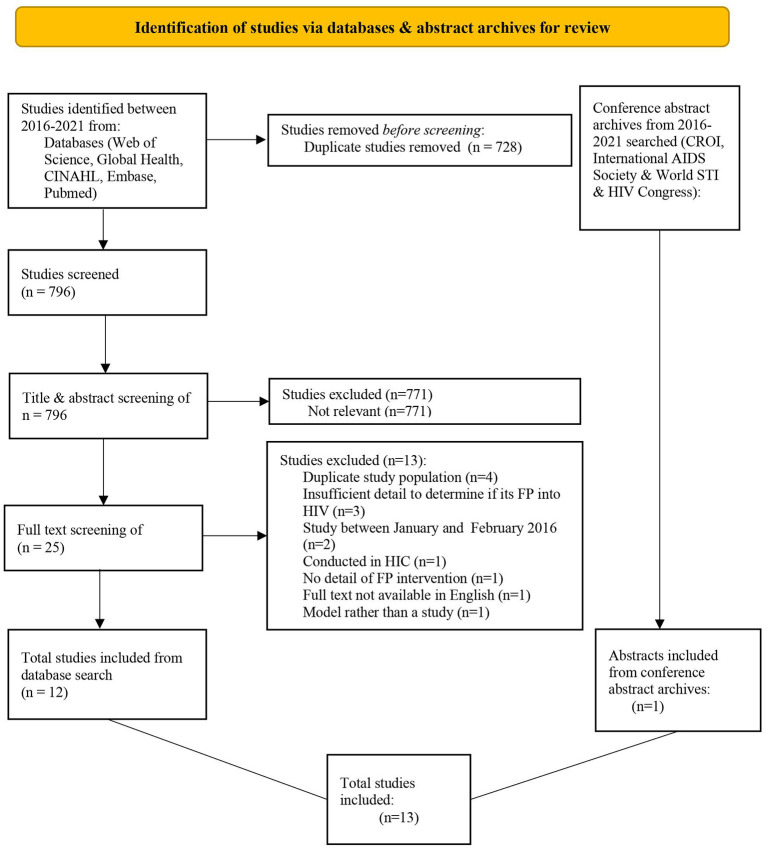
PRISMA flow chart depicting the process leading to the inclusion of studies for the review.

The database search strategy yielded 1,524 results, leaving 796 records following the deletion of duplicates (Figure 1). The remaining records were screened by title, abstract and full-text and 12 studies were deemed suitable for inclusion from the database search (11 full-text articles and one conference abstract). One additional conference abstract was identified through abstract archive searching (28). Efforts to contact the authors of the two included conference abstracts were unsuccessful; therefore, no supplementary information could be obtained (28, 29). No further studies were identified through either The Cochrane Central Register of Controlled Trials or the bibliographies of included studies.

A total of 48,587 women were enrolled across 13 studies (Table 1). Two studies were conducted in India which has experienced a concentrated HIV epidemic (29, 30). The remainder were conducted in countries in sub-Saharan Africa (11/13), which have experienced generalized HIV epidemics, including, Kenya (*n* = 3), Tanzania (*n* = 1), Rwanda (*n* = 1), Botswana (*n* = 1), South Africa (*n* = 1), Zambia (*n* = 1), Uganda (*n* = 2) and Malawi (*n* = 1). The studies included two randomized trials, two quasi-experimental studies, four before/after designs, two cross-sectional and three cohort studies. All studies except one included an element of FP counseling in their interventions and 7/13 expanded the onsite provision of contraception.

**Table 1:** Study characteristics of all included studies.

**Author, date, location**	**Study design, sample size**	**Duration (months)**	**FP intervention**	**HIV services and target**	**Outcome assessment**
Hawkins et al. (37) Location: Botswana	Design: Before/after design Sample size: 248 Intervention: 107 Comparator: 141	11 October 2017 to August 2018	FP counseling & on-site referral to contraception provider	Service: 1 HIV clinic Target population: WLHIV aged 18–45	Outcome: Contraception use Definition: Number of women expressing desire to use a LARC method Assessment: Survey to participants and providers before and after intervention.
Joshi et al. (31) Location: Mumbai, India	Design: Quasi experimental Sample size: 283 Intervention: 141 Comparator: 142	30 July 2011 to December 2013	Repeated FP counseling, posters on dual methods, referral to FP clinics.	Service: Integrated counseling & testing centers & PPTCT centers at 2 tertiary care hospitals. Target population: WLHIV	Outcome: Contraception use, unintended pregnancy, Definition: Dual method contraception uptake, number of unintended pregnancy Assessment: A semi-structured questionnaire was delivered to all enrolled women at baseline and each follow-up.
Joshi et al. (33) Location: Mumbai, India	Design: Before/after design Sample size: 556 Intervention: 256 Comparator: 300	26 April 2016 to May 2018	Contraceptive needs assessment, dual contraception counseling, referral to FP centers	Service: 2 district hospital ART centers Target population: PLHIV	Outcome: Contraception use Definition: Dual method contraception uptake Assessment: Pre-tested semi-structured interview at enrolment and follow-up.
Cohen et al. (39) Location: Kenya	Design: Cohort analysis Sample size: 17,310 Intervention: 11,628 Comparator: 5,682	34 December 2009 to October 2012	FP counseling & provision.	Service: 18 HIV clinics Target population: women receiving care at clinics	Outcome: Contraception use Definition: More effective contraception use, any contraception method use. Assessment: Patient encounter forms were initially used to gather data.
Dulli et al. (34) Location: Naivasha and Nanyuki, Kenya	Design: Quasi-experimental Sample size: 719 Intervention: 360 Comparator: 359	12 June 2013 to May 2014	Routine screening, expanded onsite availability of FP methods, dual protection education.	Service: 2 Drop in centers providing HIV counseling & testing Target population: FSW	Outcome: Contraceptive use Definition: Modern contraceptive use, dual method use Assessment: Interviews were conducted on participants.
Chen et al. (30) Location: Kenya	Design: Cross-sectional Sample size: 4,805	4 June to September 2016	FP consultation or provision of methods or both	Service: 106 HIV care & treatment centers Target population: WLHIV	Outcome: Contraceptive use, unintended pregnancy, unmet need for FP Definition: Modern contraception prevalence rate, dual method use, LARC uptake Assessment: A FP survey was delivered to all women attending the HIV centers over a 5-day period
Tweya et al. (38) Location: Lilongwe, Malawi	Design: Retrospective cohort analysis Sample size: 10,472	47 January 2013 to December 2016	Provision of FP methods	Service: ART clinic Target population: WLHIV aged 15-49	Outcome: Contraception use Definition: Any contraception method use, more effective contraception use Assessment: Routine data was collected through an EMR system after clinic consultations
Guillaine et al. (36) Location: Southern Kayonza and Kirehe, Rwanda	Design: Retrospective cohort Sample size: 185	12 July 2012 to June 2013	Combined clinic offering FP, psychosocial support & breastfeeding counseling	Service: PMTCT clinics Target population: WLHIV	Outcome: Contraception use Definition: Modern contraception use Assessment: Information on mother-infant pairs were compared from when they first attended the integrated clinic to when they had been at the clinic for 12 months. Data was collected as part of routine clinical care.
Mantell et al. (32) Location: Cape Town, South Africa	Design: Randomized intervention trial Sample size: 214 Intervention: 104 Comparator: 110	NR	Contraceptive method provision, safer conception methods & types of contraception counseling, referrals for pregnancy termination.	Service: 4 HIV clinics Target population: Newly diagnosed PLHIV	Outcome: Contraception use, safer conception Definition: Dual method use, adherence to safer conception guidelines Assessment: Face-to-face interviews
Casalini et al. (28) Location: Tanzania	Design: Before/after design Sample size: 9,332	5 August to December 2015	FP counseling & services	Service: Mobile community based HIV testing and counseling ‘'plus” Target population: FSW	Outcome: Contraception use Definition: Any contraception method use Assessment: Patient data was obtained during community testing and counseling services and entered into a central database
Nabirye et al. (35) Location: Uganda	Design: Cross-sectional Sample size: 2,760	4 August to November 2016	FP counseling	Service: 245 HIV clinics, PMTCT care Target population: WLHIV	Outcome: Contraception use Definition: Modern contraception use Assessment: Data collated from a national cross-sectional survey
Wagner et al. (24) Location: Wakiso, Masaka, Mbale, Jinja, Rukugiri, Mbarara, Uganda	Design: Three-arm cluster randomized intervention trial Sample size: 390 Intervention: 260 Comparator: 130	12	Safer conception counseling or support for modern contraceptive use.	Service: 6 HIV clinics Target population: HIV+ clients considering childbearing with an HIV- partner	Outcome: Contraception use, safer conception Definition: Modern contraception use among those not trying to conceive, used timed condom-less sex or manual self-insemination accurately. Assessment: Participants were interviewed using computer assisted software at each time point
Medley et al. (29) Location: Lusaka, Zambia	Design: Before/after design Sample size: 1,313 Intervention: 629 Comparator: 684	15 April 2018 to June 2019	Full range of FP methods, safer conception counseling, facilitated referral for refills	Service: 6 HIV clinics Target population: WLHIV	Outcome: Contraception use, unmet need for FP, safer conception Definition: Effective contraception use, dual contraception use, discussed safer pregnancy with a provider. Assessment: Different participants interviewed pre- and post-intervention and data extracted from their medical records

*FP, Family planning; HIV, human immunodeficiency virus; WLHIV, women living with HIV; LARC, long acting reversible contraception; PPTCT, prevention of parents-to-child transmission; PMTCT, prevention of mother-to-child transmission; DIC, drop in centre; FSW, female sex workers; PLHIV, people living with HIV; ART, antiretroviral therapy*.

### Associations Between FP-HIV Integration and Contraceptive Uptake

All studies examined the effect of integration on contraceptive uptake with the most frequently used categories being dual method and modern contraceptive use (Table 2). Out of the six studies investigating dual method contraceptive use, five suggested an association between integration and increased dual method use (29–33). Chen et al., a cross-sectional study, found 30% of individuals attending non-integrated facilities use dual methods compared to 40% at integrated services (*p* < 0.01) (30). A quasi-experimental study design conducted by Joshi et al. demonstrated that after repeated FP counseling, poster advertisement and a referral mechanism was put in place, uptake of dual contraception was 32.6% in the intervention arm compared to 10.6% in the control group (31). Medley et al. similarly found dual contraceptive use was 9% pre-intervention compared to 18% post-intervention (29). Joshi et al. reported 0% dual contraception uptake pre-intervention compared to 44.6% at the endpoint (33). Mantell et al. observed a 10% absolute increase in use of dual contraceptives in the intervention group compared to the control (32). Dulli et al., found no association between integrated facilities and dual method contraception (34). After introducing the enhanced integration intervention to the experimental arm, Dulli et al. reported an adjusted odds ratio (aOR) of 0.76 (95% CI: 0.69–1.37) for dual method use (34).

**Table 2:** Associations between integrated FP-HIV services and contraception use.

**Study**	**Study design**	**Assessment**	**Results**
Hawkins et al. (37) Location: Botswana	Before/after design	Survey to participants and providers before and after intervention.	Desire to use LARC: 29% (31/107) (95% CI: 20.40–37.60) of women expressed desire to use LARC post-intervention compared to 6% (8/141) (95% CI: 2.08–9.92) pre-intervention (*p* < 0.001)
Joshi et al. (31) Location: Mumbai, India	Quasi experimental	A semi-structured questionnaire was delivered to all enrolled women at baseline and each follow-up.	Dual method contraception uptake: 32.6% (46/141) (95% CI: 24.9–40.3) of individuals were using dual methods in the intervention group by the end of the study compared to 10.6% (15/142) (95% CI: 5.5–15.7) in the control group (*p* < 0.05)
Joshi et al. (33) Location: Mumbai, India	Before/after design	Pre-tested semi-structured interview at enrolment and follow-up.	Dual method contraception uptake: 44.6% (248/556) (95% CI: 40.46–48.74) of individuals were using dual methods post-intervention compared to 0% (0/556) pre-intervention.
Cohen et al. (39) Location: Kenya	Cohort analysis	Patient encounter forms were initially used to gather data.	More effective contraception use: 44.2% were using more effective contraceptive methods post-intervention compared to 31.7% at baseline *p* < 0.0001, PR: 1.39 (95% CI: 1.19–1.63) Any contraception method use: 79.5% were using any contraceptive method post-intervention compared to 70.5% at baseline *p* = 0.09, PR: 1.13 (95% CI: 0.94–1.35)
Dulli et al. (34) Location: Naivasha and Nanyuki, Kenya	Quasi-experimental	Interviews were conducted on participants.	Modern contraception use: The odds of individuals in the intervention group using modern contraception were 1.38 times higher than in the control group (OR: 1.38 95% CI: 1.04–1.83) Dual method use: The odds of individuals in the intervention group using dual methods were 0.76 than of in the control group (OR: 0.76 95% CI: 0.69–1.37) This was adjusted for education and age
Chen et al. (30) Location: Kenya	Cross-sectional	A FP survey was delivered to all women attending 106 HIV centers over a 5-day period and then integrated facility statistics were compared to non-integrated facility statistics.	Modern contraception prevalence rate: 88% of respondents attending integrated facilities used modern contraception compared to 80% at non-integrated facilities (*p* < 0.01) Dual method use: 40% of respondents attending integrated facilities used dual methods compared to 30% at non-integrated facilities (*p* < 0.01) LARC uptake: 28% of respondents attending integrated facilities use LARC methods compared to 20% at non-integrated facilities (*p* < 0.001)
Tweya et al. (38) Location: Lilongwe, Malawi	Retrospective cohort analysis	Routine data was collected through an EMR system after clinic consultations	More effective contraceptive use: 27% (2,015/7,463) (95% CI: 26.00–28.00) of individuals were using more effective contraceptive methods at the endpoint compared to 26% (413/1,590) (95% CI: 23.84–28.16) at baseline Any contraceptive method use: 62% (4,627/7,463) (95% CI: 60.90–63.10) of individuals were using any method of contraception at the endpoint compared to 28% (445/1,590) (25.79–30.21) at baseline
Guillaine et al. (36) Location: Southern Kayonza and Kirehe, Rwanda	Retrospective cohort	Information on mother-infant pairs were compared from when they first attended the integrated clinic to when they had been at the clinic for 12 months. Data was collected as part of routine clinical care.	Modern contraception use: 72% (131/182) (95% CI: 65.47–78.53) of participants were using modern contraception methods post-intervention compared to 30.3% (57/185) (95% CI: 23.68–36.92) at enrolment
Mantell et al. (32) Location: Cape Town, South Africa	Randomized intervention trial	Face-to-face interviews at baseline, follow up 1 and follow up 2.	Dual method use: The intervention group saw a 22% increase in use of dual method from baseline to follow-up 2. The control group observed a 12% increase from baseline to follow-up 2.
Casalini et al. (28) Location: Tanzania	Before/after design	Patient data was obtained during community testing and counseling services and entered into a central database	Any contraceptive method use: 35% (949/2,691) (95% CI: 33.20–36.80) of participants were using a contraceptive method post-intervention compared to 22% (592/2,691) (95% CI: 20.43–23.57) at baseline.
Nabirye et al. (35) Location: Uganda	Cross-sectional	Data collated from a national cross-sectional survey	Modern contraception use: The prevalence of modern contraception use in integrated facilities was 1.21 times higher than in non-integrated services (adjusted PR: 1.21 95% CI: 1.10–1.33 *p* < 0.001)
Wagner et al. (34) Location: Wakiso, Masaka, Mbale, Jinja, Rukugiri, Mbarara, Uganda	Three-arm cluster randomized intervention trial	Participants were interviewed using computer assisted software at each time point	Modern contraception use among those not trying to conceive: 14.6% (7/48) (95% CI: 4.60–24.60) of participants were using modern contraceptives in the intervention group compared to 19.4% (12/61) (95% CI: 9.48–29.32) in the control group p=0.487. Adjusted OR: 3.72 (95% CI: 0.37–37.49)
Medley et al. (29) Location: Lusaka, Zambia	Before/after design	Different participants were interviewed pre- and post-intervention and data extracted from their medical records	Effective contraception use: 49% (195/402) (95% CI: 44.12–53.88) of participants used effective contraception post-intervention compared to 38% (133/379) (95% CI: 33.12–42.88) pre-intervention p=0.003 Dual contraception use: 18% (73/402) (95% CI: 14.24–21.76) of participants used dual contraception post-intervention compared to 9% (30/379) (95% CI: 6.12–11.88) pre-intervention *p* = 0.0003

*FP, family planning; HIV, human immunodeficiency virus; LARC, long-acting reversible contraception; PR, prevalence ratio; OR, odds ratio*.

Results of four studies suggest an association between integrated services and increased use of modern contraception methods (30, 34–36). Chen et al. observed integrated facilities have a modern contraception prevalence rate of 88% compared to 80% at non-integrated facilities (30). Dulli et al. also presented an aOR of 1.38 (95% CI: 1.04–1.83), suggesting an association between integrated facilities and increased use of modern contraception (34). Additionally, an adjusted prevalence ratio (PR) of 1.21 (95% CI: 1.10–1.33 *p* < 0.001) reported by Nabirye et al. indicates an association between receiving FP counseling and increased use of modern contraception (35). Guillaine et al. also reported an increase in modern contraception use from baseline to post-intervention (30.3–72%, respectively) (36). Conversely, Wagner et al. found no evidence of integration increasing modern contraceptive use with an aOR of 3.72 (95% CI: 0.37–37.48) when comparing their high intensity intervention to the low intensity intervention. However, this had limited power due to a small sample size (24).

Hawkins et al. reported on women's desire for wanting to utilize LARC pre-intervention compared to post-intervention (37). A positive association between integration and more positive attitudes toward FP was identified with the desire to use LARC at 6% at pre-intervention compared to 29% at the endpoint (*p* < 0.001) (37).

### Associations Between FP-HIV Integration and Unmet Need for FP

Two studies appraised this outcomes, both of which provide evidence for integration reducing the unmet need for FP (Table 3). Chen et al. reported integrated facilities had an unmet need of 8% compared to non-integrated facilities having an unmet need of 15% (30). Lastly, Medley et al. observed a reduction from 59% pre-intervention to 46% post-intervention (29). This remained significant after adjustment for facility, age group and time since diagnosis.

**Table 3:** Associations between integrated FP-HIV services and unmet need for FP.

**Study**	**Study design**	**Assessment**	**Results**
Chen et al. (30) Location: Kenya	Cross-sectional	A FP survey was delivered to all women attending 106 HIV centers over a 5-day period and then integrated facility statistics were compared to non-integrated facility statistics.	Unmet need for modern FP methods: 15% of women attending non-integrated facilities had an unmet need for modern FP compared to 8% in integrated facilities (*p* < 0.01)
Medley et al. (29) Location: Lusaka, Zambia	Before/after design	Different participants were interviewed pre- and post-intervention and data extracted from their medical records	Unmet need for FP: 46% (184/402) (95% CI: 41.12–50.88) of women in the post-intervention group had an unmet need for FP compared to 59% (210/379) (95% CI: 54.04–63.96) in the pre-intervention group (*p* = 0.0003)

*FP, family planning; HIV, human immunodeficiency virus*.

### Associations Between FP-HIV Integration and Safer Conception

Three studies assessed the effect of integrated services on safer conception methods for WLHIV desiring a pregnancy (Table 4) (24, 29, 32). Medley et al. included safer pregnancy counseling in their FP services and consequently observed a 12% absolute increase in women discussing safer pregnancy with their HIV provider (29). Wagner et al. introduced safer conception counseling into HIV care and subsequently observed a 24.1% increase in the correct use of timed condom-less sex or manual self-insemination in the intervention arm compared to usual care accompanied by a covariate aOR of 91.84 (95% CI: 4.94–1709 *p* < 0.01) (24). Mantell et al. assessed a binary outcome reporting whether safer conception guidelines were being adhered to (32). The result was deemed significant if the difference between the enhanced intervention and standard of care groups was >10%. Mantell et al. observed a difference in adherence to safer conception guidelines of 11% (95% CI −0.04, 0.27) between the study groups, thus meeting this criterion (32). However, this was not statistically significant.

**Table 4:** Associations between integrated FP-HIV services and safer conception.

**Study**	**Study design**	**Assessment**	**Results**
Mantell et al. (32) Location: Cape Town, South Africa	Randomized intervention trial	Face-to-face interviews at baseline, follow up 1 and follow up 2.	Following safer conception guidelines: 83% followed safer conception guidelines in the intervention group compared to 72% in the control group (*p* = 0.58)
Wagner et al. (24) Location: Wakiso, Masaka, Mbale, Jinja, Rukugiri, Mbarara, Uganda	Three-arm cluster randomized intervention trial	Participants were interviewed using computer assisted software at each time point.	Used timed condom-less sex or manual self-insemination accurately: 24.1% (46/191) (95% CI: 18.04–30.16) used methods accurately in the intervention group compared to 0% (0/85) in the control group (OR: 91.84 95% CI: 4.94–1709) *p* < 0.01 This was adjusted for site, age, sex, education, time since HIV diagnosis
Medley et al. (29) Location: Lusaka, Zambia	Before/after design	Different participants were interviewed pre- and post-intervention and data extracted from their medical records	Discussed safer pregnancy with a healthcare provider: 39% (97/249) (95% CI: 32.94–45.06) in the post-intervention group discussed safer pregnancy compared to 27% (56/210) (95% CI: 21.00–33.00) in the pre-intervention group (*p* = 0.0093)

### Associations Between FP-HIV Integration and Unintended Pregnancy

Two studies assessed the association between integrated facilities and unintended pregnancy (Table 5) (30, 31). Chen et al. found no difference in the proportion of unintended pregnancy in non-integrated facility settings (35%) compared to integrated services (36%) (*p* = 0.81) (30). Joshi et al. reported the number of unintended pregnancies in the intervention and control groups as 13 and 20, respectively (31). Three studies looked at pregnancy incidence, however, in the absence of information regarding if these pregnancies were planned or not, interpreting these figures is challenging.

**Table 5:** Associations between integrated FP-HIV services and unintended pregnancy.

**Study**	**Study design**	**Assessment**	**Results**
Joshi et al. (31) Location: Mumbai, India	Quasi experimental	A semi-structured questionnaire was delivered to all enrolled women at baseline and each follow-up.	Number of unintended pregnancies: There were 13 unintended pregnancies recorded in the intervention group (n=150) (95% CI: 4.41–13.59) compared to 20 in the control group (n=150) (95% CI: 7.61-18.39) p>0.05
Chen et al. (30) Location: Kenya	Cross-sectional	A FP survey was delivered to all women attending 106 HIV centers over a 5-day period and then integrated facility statistics were compared to non-integrated facility statistics.	Last pregnancy was unintended: 36% of women at integrated facilities reported their previous pregnancy was unintended compared to 35% at non-integrated facilities (p=0.81)

*FP, family planning; HIV, human immunodeficiency virus*.

### Quality Assessment

The median score after applying the 9-point quality scale was 3 out of 9 with a mean of 3.5 making it no different from previous reviews (Table 6). Using the pre-defined criteria, 2/13 were classified as high-quality studies, 3/13 were considered to be at risk of bias and 8/13 were weak quality studies.

**Table 6:** Quality analysis of included studies employing the 9-point quality scale.

**Study**	**Pre/post-intervention data available**	**Control or comparison group**	**Cohort**	**Comparison groups equivalent at baseline on characteristics**	**Comparison groups equivalent at baseline on outcome measures**	**Random assignment**	**Participants randomly selected for assessment**	**Control for confounders**	**Follow up rate ≥75%**	**Total score out of 9**
Casalini et al. (28)										1
Chen et al. (30)										1
Cohen et al. (39)										3
Dulli et al. (34)										5
Guillaine et al. (36)										3
Hawkins et al. (37)										1
Joshi et al. (33)										3
Joshi et al. (31)										6
Mantell et al. (32)										7
Medley et al. (29)										4
Nabirye et al. (35)										2
Tweya et al. (38)										2
Wagner et al. (34)										7
				Key						
					Yes					
					No					
					Not applicable					
					Not reported					

The Newcastle-Ottawa scale was used to assess the quality of the three cohort studies. All three studies achieved scores ranging from 4 to 5 and were considered to be at high risk of bias. Tweya et al. achieved a score of 4, Guillaine et al. a score of 5 and Cohen et al. a score of 5 (36, 38, 39). Tweya et al. and Guillaine et al. did not contain a non-exposed cohort and therefore the selection of a non-exposed cohort was denoted as ‘not applicable' (36, 38).

## Discussion

This systematic review suggests that the integration of FP with HIV services improves uptake of and unmet need for FP among WLHIV in LMICs. The review builds on the evidence from previous systematic reviews with regard to contraceptive uptake. While the first two reviews conducted by Spaulding et al. and Wilcher et al. did find some association between integrated services and increased general contraceptive use, they did not report on specific types of contraceptive use (18, 19). Haberlen et al., however, broke down contraceptive use into subsections including modern contraception and dual method use (17). The current review reinforces the finding of Haberlen et al. that integration increases modern contraception use (17).

There was evidence that integration increased dual method use, building on the evidence presented in the review by Haberlen et al. (17). Despite this evidence, in general, dual contraception use was lower than modern contraceptive use. Chen et al. reported that in integrated facilities only 40% of individuals employed dual contraception, despite a modern contraception prevalence rate of 88% (30). The reason for low uptake of dual contraception may be simply an unwillingness to use condoms or to a lack of awareness of the importance of employing condoms in addition to other methods to prevent the acquisition of STIs and HIV. It may also be attributed to gender inequality, specifically unequal gender power dynamics within relationships. While modern contraception forms allow for increased autonomy, frequently, a women's ability to negotiate condom use is limited, particularly in vulnerable groups such as female sex workers (40). It is also important to note that self-reported condom use poses a risk of social-desirability bias, potentially inflating the results for this outcome (41).

This review presents accumulating evidence of an effect of integration decreasing the unmet need for FP. The previous review conducted by Haberlen et al., found three studies evaluated the impact of integration on unmet need (17). Two of these suggested an association between integration and decreased unmet need for FP (42, 43). This was not enough evidence to conclude an effect. However, the two studies from the previous review combined with Chen et al. and Medley et al. in this review, provides further evidence suggesting that integration does decrease the unmet need for FP (29, 30).

In line with previous systematic reviews, there remains insufficient data to conclude an effect on unintended pregnancy. Only Chen et al. and Joshi et al. reported on unintended pregnancy and both reported integration had no effect (30, 31). The limited reporting on this outcome is likely due to this being hard to measure. Nevertheless, contraceptive uptake, unmet need for FP and unintended pregnancy are intrinsically linked. If providing integrated care likely increases modern and dual contraception use while decreasing the unmet need for FP, as this review shows, this is anticipated to lead to a reduction in unintended pregnancies.

Despite not being able to draw a conclusion on safer conception due to limited data, the review included the first two randomized intervention trials assessing this outcome, Mantell et al. and Wagner et al. (24, 32). These high-quality studies reported increases in the use of safer conception methods in their intervention arms compared to the control, however, the finding of Mantell et al. was not statistically significant (32).

Moving forward, this review has revealed the need for further research on the effectiveness and impact of incorporating safer conception interventions for WLHIV within HIV services. Bringing to an end outdated beliefs surrounding WLHIV not being able to safely conceive and interventions that teach and support WLHIV to safely conceive will aid in reducing stigma and empowering PLHIV.

We acknowledge several limitations. Limited information was available from conference abstracts. Attempts to obtain further information from the first authors of the two conference abstracts were unsuccessful. Therefore, some detail regarding methodologies were missing potentially leading to a downgrading of the quality of these studies. Selection and screening of studies was conducted by only one reviewer which may have led to some studies being missed. The outcomes were obtained through self-report which may introduce social desirability bias. Publication bias can also not be excluded. As in the previous reviews, the overall quality of studies remained low and there was substantial heterogeneity across studies, precluding a meta-analysis.

Notwithstanding these limitations, this review builds on existing evidence demonstrating the benefits of integration of FP into HIV services for WLHIV in terms of enhancing modern and dual contraceptive uptake and reducing the unmet need for FP. Therefore, focus should now be on translating the evidence accumulated over the past two decades into practice in LMICs. This review has highlighted the gaps in the research, specifically the scarcity of integrated programmes focusing on safer conception for WLHIV desiring a pregnancy and the importance of such programmes.

## Data Availability Statement

The original contributions presented in the study are included in the article/supplementary material, further inquiries can be directed to the corresponding author/s.

## Author Contributions

TG-M and RF conceived the study. TG-M conducted the systematic search, extracted the data, and drafted the manuscript. RF and KK edited the full manuscript. All authors contributed to the article and approved the submitted version.

## Conflict of Interest

The authors declare that the research was conducted in the absence of any commercial or financial relationships that could be construed as a potential conflict of interest.

## Publisher's Note

All claims expressed in this article are solely those of the authors and do not necessarily represent those of their affiliated organizations, or those of the publisher, the editors and the reviewers. Any product that may be evaluated in this article, or claim that may be made by its manufacturer, is not guaranteed or endorsed by the publisher.
